# Confocal Raman imaging reveals the impact of retinoids on human breast cancer via monitoring the redox status of cytochrome *c*

**DOI:** 10.1038/s41598-023-42301-z

**Published:** 2023-09-12

**Authors:** Jakub Maciej Surmacki, Halina Abramczyk

**Affiliations:** Laboratory of Laser Molecular Spectroscopy, Institute of Applied Radiation Chemistry, Faculty of Chemistry, Lodz University of Technology, Wroblewskiego 15, 93-590 Lodz, Poland

**Keywords:** Imaging, Raman spectroscopy, Cancer

## Abstract

This paper expands the current state of knowledge on impact of retinoids on redox status of cytochrome *c* in cancers. Little is known how the expression of cytochromes may influence the development of cancers. We studied the effect of the redox status of the central iron ion in heme of cytochrome *c*. We determined the redox status of the iron ion in cytochrome *c* in mitochondria, cytoplasm, lipid droplets, and endoplasmic reticulum of the human breast cancer cells by Raman imaging. We incubated human breast adenocarcinoma cells (SK-BR-3) with retinoic acid, retinol and retinyl ester (palmitate) at concentration of 50 μM for 24 h. We recorded the Raman spectra and images of human breast cancer in vitro SK-BR-3 cells receiving redox stimuli by retinoic acid, retinol and retinyl ester (palmitate). The paper provides evidence that retinoic acid and retinol are pivotally important for mitochondrial energy homeostasis by controlling the redox status of cytochrome *c* in the electron transport chain controlling oxidative phosphorylation and apoptosis. We discussed the role of retinoids in metabolism and signaling of cancer cells. The paper provides experimental support for theoretical hypothesis how retinoic acid/retinol catalyse resonance energy transfer reactions and controls the activation/inactivation cycle of protein kinase PKCδ.

## Introduction

Retinoids play an important role in cell signaling. Abnormal retinoid signaling in the cytoplasm and nucleus has been reported in human cancers^1^. It has been reported that retinoic acid induces cell death via targeting mitochondria^2^. However, the precise mechanism of mitochondrial engagement in retinoic acid-induced apoptosis remains vague.

Recently we demonstrated that label-free Raman microscopy helps to clarify of the precise role of retinoids in the metabolism and signaling of cancer cells. We examined the role of retinoids in human cell lines of normal astrocytes (NHA), high-grade tumor cells of glioblastoma (U-87 MG) and in human medulloblastoma and glioblastoma tissues^3^.

The role of retinoids in cell signaling is closely related to retinoid-binding proteins as retinoids are not found as free molecules in extracellular matrix. Instead, they are associated with binding proteins for delivery to target tissue, usually over very short distances. There are three main families of retinoid-binding proteins: retinol-binding protein (RBP), cellular retinol (or retinal)-binding protein CRBP, and cellular retinoic acid-binding protein (CRABP). RBP binds to retinol and its synthesis takes place not only in the liver but also in heart, testis, eyes, spleen and other organs. Because the liver stores vitamin A, the expression and release of RBP–retinol complex will depend on the availability of vitamin A. It has been demonstrated that under vitamin A deficiency, RBP expression is downregulated and its release to circulation is inhibited^4,5^. Vitamin A is involved in cell signaling by attachment to the retinol-binding protein receptor termed STRA6 (stimulated by retinoic acid (RA))^6^.

STRA6 is a ligand-activated transmembrane cell surface protein that functions as a receptor for a family of retinol-binding proteins (RBPs) that plays a role as carrier protein for retinol. Upon binding of the retinol to RBP (also termed the holo-RBP or RBP–ROH complex) STRA6 activates JAK/STAT signalling to transfer information from the extracellular environment to the nucleus, resulting in DNA transcription and inducing the expression of target genes involved in many processes such as immunity, proliferation, differentiation, and apoptosis. Disrupted JAK/STAT signaling may lead to a variety of diseases, such as cancers and disorders of the immune system^7^. Janus kinases (JAKs) belong to an enzyme family of intracellular, nonreceptor tyrosine kinases that activate STAT signaling. STAT (signal transducer and activator of transcription) is a member of protein family that activates genes through transcription. The transport of retinol to the interior of the cell occurs via the retinol acceptor CRBP1 protein attached to the CRBP binding loop of STRA6. This event activates the intracellular non-receptor tyrosine kinase enzyme JAK2, which transfers a phosphate group from ATP to a protein in the cell, thereby phosphorylating STRA6 at Y643 (tyrosine)^8^. The detailed mechanism of JAK2 activation by the retinol–RBP complex is still unknown and awaits further investigation to elucidate the cascade of downstream signals and events necessary to activate (or suppress) gene transcription in the nucleus.

**Figure 1:**
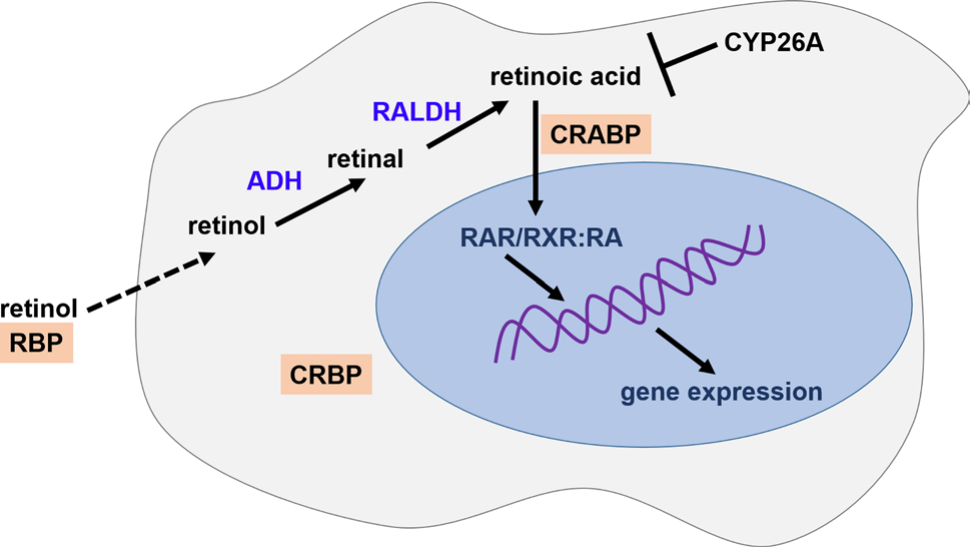
Retinoic acid metabolism.

Generally, to regulate gene expression, all-trans- and 9-cis-RA bind to nuclear receptors, which act as ligand-induced transcription factors to bind to specific sequences in the DNA and modulate the transcription of target genes (Fig. 1). It should be mentioned that retinoic acid (RA) may bind to RA receptors (RARs) or to retinoic X receptors (RXRs), both of them belonging to the steroid/thyroid/vitamin D receptor super family^9^. Figure 1 shows retinoic acid metabolism. Retinol gives rise to retinoic acid through the activity of different families of enzymes, including RALDH, which catalyzes the last step in an irreversible manner. Once in the cytoplasm, RA binds to CRABP and is transferred to the nucleus, where it is recognized by the nuclear receptors (RAR/RXR), and it binds to the DNA to regulate gene expression^9^.

There is growing evidence that retinoic acid is a key player in immunity^9^. It has been shown that RA induced genes (RIG-I) trigger a signaling-abortive anti-SARS-CoV-2 defence in human lung cells^10^. It is well known that the immune system is critical in fighting cancer Numerous studies demonstrate that RA may also play a role in regulating the immune response to tumors^11,12^. However, impact of retinoic acid on immune cells, inflammatory diseases and cancer is still unknown. The potential of RA on immune responses raises many questions on how β-carotene–retinol metabolism affects cancer disease. There is a significant progress in development of immunotherapy drugs that activate innate immune cells, and drugs that activate T-cells in the adaptive immune system. The immune system relies on receptor proteins on the immune cells to detect the invading viruses or bacteria. Unfortunately, cancer cells don't trigger an immune response because they are the body’s own cells that have mutated and no longer behave like normal cells. That is why the immune system doesn’t recognize the distinction, these dangerous cancer cells can continue to grow, divide and spread throughout the body. This new mode of RIG-I recognition does not stimulate its ATPase, thereby aborting the activation of the conventional mitochondrial antiviral-signaling protein-dependent pathways, which is in accordance with lack of cytokine induction.

RA may enhance protective antitumor immunity through mechanisms such as induction of cell differentiation and enhancement of migration to lymph nodes. On the other side, RA shows aborting the activation of the conventional mitochondrial signaling protein-dependent pathways, which decreases immunity in cancers.

To understand the role of retinoids in cellular signal transduction in cancers we must use proper tools for sensing retinoids in vivo to monitor retinoid distribution and temporal dynamics in cells and tissues. Raman spectroscopy and imaging are an excellent tool that can not only provides biochemical profile of cells and issues but also understanding of the disease as it progresses.

Understanding the metabolism of retinoids in normal and cancer cells is still very limited because so far there were no experimental methods available to track retinoids in vivo in specific organelles in living cells. The Raman-based method we present in this paper provides an excellent tool to extend our knowledge in this field.

To answer the questions on the role of retinoids in metabolism and signaling of cancer cells we recorded the Raman spectra of cells receiving redox stimuli by retinoic acid (RA), retinol and retinyl palmitate in vitro cell cultures. For these purposes, we incubated the breast cancer cells (SK-BR-3) with retinoic acid, retinol and retinyl ester (palmitate) at concentration of 50 μM for 24 h of incubation.

## Results

### Retinoids in cancers studied by Raman imaging

To clarify the role of retinoids in cancer we studied the breast cancer cells in vitro by Raman imaging. They represent the human epithelial breast adenocarcinoma SK-BR-3 cell line, which overexpresses the HER2/c-erb-2 gene. We incubated the breast cancer cells with retinoic acid, retinol and retinyl ester (palmitate). Raman spectra of retinol, retinoic acid, retinyl palmitate, oxidized and reduced form of cytochrome *c* are presented on Supplementary Figure 1.

**Figure 2:**
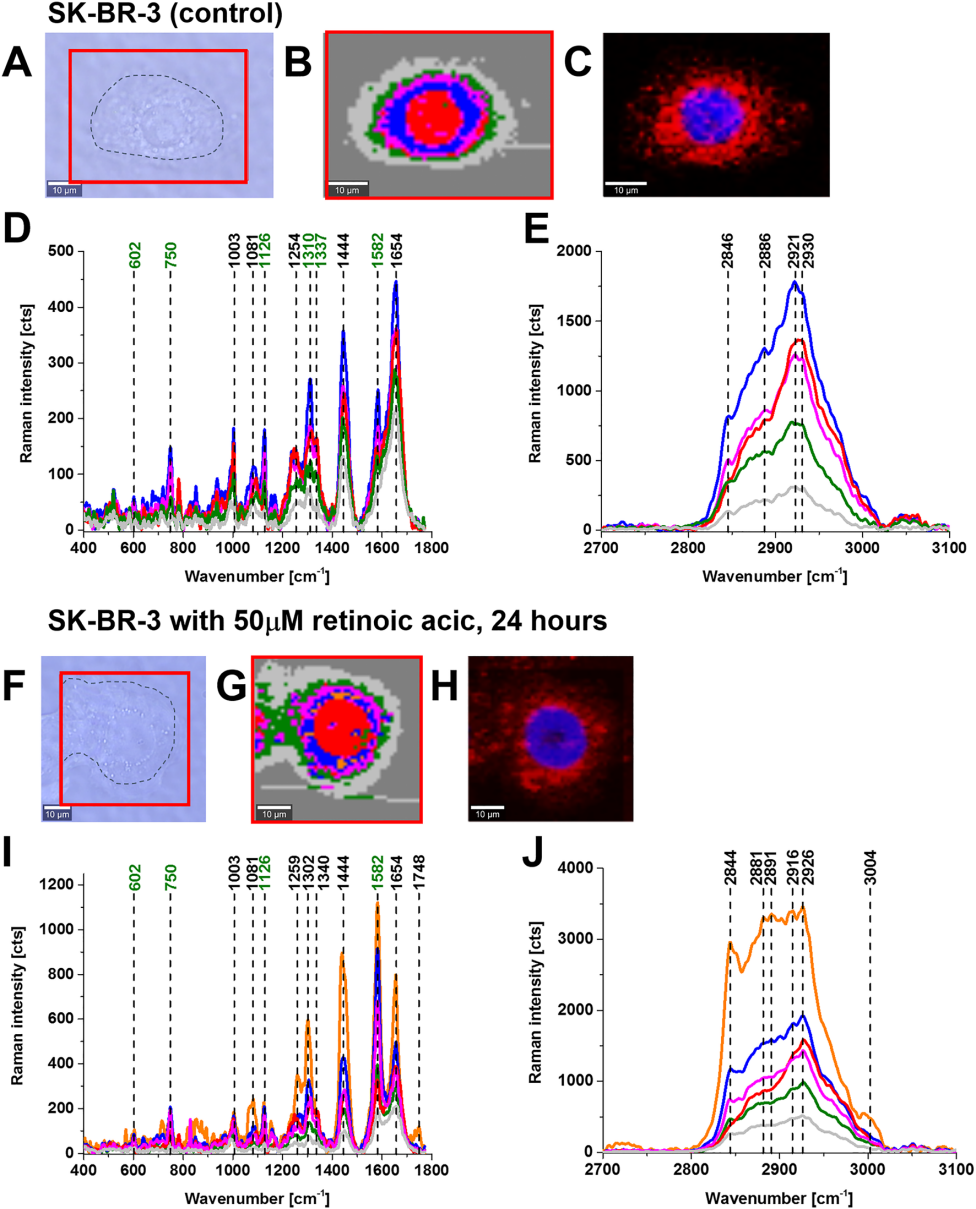
Raman imaging of a typical breast cancer cell (SK-BR-3) without (control) and incubated with retinoic acid (50 μM) for 24 h. Microscopy images (**A**,**F**), Raman images with the respective Raman spectra (**B**,**D**,**E** and **G**,**I**,**J**) of a nucleus (red), lipid droplets (orange) and endoplasmic reticulum (blue), cytoplasm (green), mitochondria (magenta), cell border (light grey). Fluorescence images (**C**,**H**). Resolution of Raman images 1 μm, integration time 0.3 s, 10 mW at 532 nm and resolution of fluorescence images 1 μm, integration time 0.01 s (Nucleus—blue (Hoechst 33342), ER/lipid droplets—red (Oil Red O)).

Figure 2 shows a typical breast cancer control cell and a cell incubated with retinoic acid (50 μM) for 24 h monitored by microscopy image, Raman image, and fluorescence images. The Raman image was obtained by applying Cluster analysis (described in the “Methods” section). The colours in the Raman image correspond to the specific organelles inside the cell: red colour corresponds to the nucleus, blue colour—to endoplasmic reticulum, orange colour—lipid droplets, magenta—mitochondrion, green—cytoplasm, light grey—cell border.

One can see that the organelles identified by the vibrations of biomolecules in the Raman imaging (Fig. 2B,G and Supplementary Figure 2) are identical with the images obtained by the other techniques such as microscope image with red oil staining (Fig. 2C,H) for lipid droplets and endoplasmic reticulum, the fluorescence image with red oil staining for lipid droplets and endoplasmic reticulum (Fig. 2B,G) and fluorescence image with Hoechst 33342 staining for nucleus (Fig. 2C,H). Figure 1D,E,I,J shows the respective Raman spectra for each organelle inside the cell.

Comparing Fig. 2D,E and Fig. 2I,J one can see many significant differences in Raman spectra for the control cells (without retinoic acid) and those incubated with retinoic acid. Detailed inspection shows a dramatic increase in the Raman signals corresponding to cytochrome *c*^11–13^: 750, 1126, 1310, 1582 cm^−1^ upon incubation with retinoic acid.

Particularly strong enhancement upon incubation with retinoic acid is observed for the ν_19_ band at 1582 cm^−1^. The band corresponds to the methine bridge vibration of cytochrome *c*. The vibrations of cytochrome *c* are clearly visible in Fig. 2 due to the Resonance Raman effect which is a remarkably effective probe to study heme proteins at in vitro cell cultures, tissues and in vivo conditions^14–17^. At non-resonant conditions the vibrations of cytochrome *c* (present at approx. 1 μM in the IMS (intramembrane space of mitochondrion) or even less (nM))^18,19^ are hidden by stronger signals of other proteins, lipids and DNA. However, at the laser excitations corresponding to the electron resonances of the Soret or Q band transitions the Raman signal increases a few orders and the Raman spectrum of cells and tissues are dominated by heme protein vibrations. In our measurements we use laser excitation at 532 nm, which is in resonance with the Q transition.

We showed that the intensity of the Raman band ν_19_ of cytochrome *c* is sensitive to the redox status of iron ion. The reduced cytochrome (Fe^2+^) has much higher intensity of the Raman bands than the oxidized cytochrome *c* (Fe^3+^)^13^. Therefore, the Raman signal increase at 1582 cm^−1^ upon incubation with retinoic acid indicates that there is shift from the oxidized cytochrome *c* to the reduced one. The change of the balance between Fe^3+^ and Fe^2+^ in the electron transport chain can activate or inhibit important physiological functions such as oxidative phosphorylation and apoptosis. The change of the redox status of cytochrome *c* in mitochondria from the oxidized form to the reduced form has very serious consequences resulting in inhibition of activation apoptosis and cytokine induction. Therefore, cytochrome *c* cannot play any longer the role of a universal DAMP (damage-associated molecular pattern) able of alarming the immune system of danger in any type of cells or tissue by aborting the activation of the conventional mitochondrial signaling pathways. The inhibition of these signaling pathways may have serious consequences on cancer development. We showed the correlation between breast and brain cancer aggressiveness and concentration of cytochrome *c*^3,13^.

**Figure 3:**
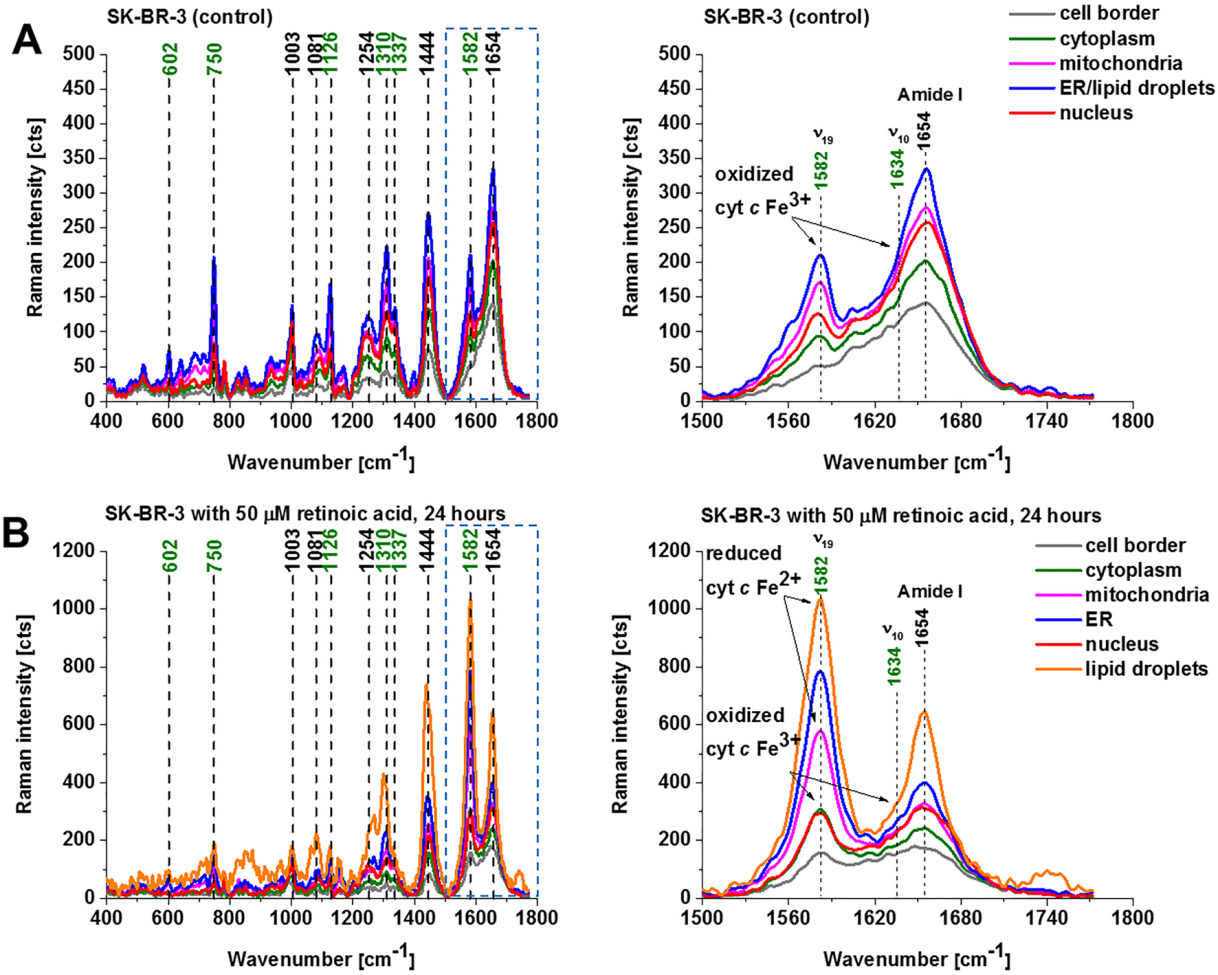
Average Raman spectra of breast cancer cell (SK-BR-3) at the 532 nm wavelength excitation in control cell (without retinoic acid) (**A**) and incubated with retinoic acid (50 μM) for 24 h (**B**). Nucleus (red), lipid droplets (orange), endoplasmic reticulum (blue), cytoplasm (green), mitochondria (magenta), cell border (light grey).

Figure 3 shows the average Raman spectra of human cancer breast cells (SK-BR-3) (control without retinoic acid) and incubated with retinoic acid (50 μM) of nucleus, mitochondria, lipid droplets/endoplasmic reticulum, cytoplasm, cell border. Figure 3 shows that the Raman band of cytochrome *c* at 1582 cm^−1^ is very strong in the cancer breast cells when incubated with retinoic acid at 532 nm excitation in contrast to the control cancer breast cells without supplementation. Therefore, it is evident that retinoic acid changes the redox status of cytochrome *c* from the oxidized (Fig. 3A) to reduced form (Fig. 3B) in mitochondria, lipid droplets and endoplasmic reticulum. In cytoplasm, nucleus, cell border cytochrome *c* is in the oxidized form both in the control cells and those incubated with retinoic acid.

**Figure 4:**
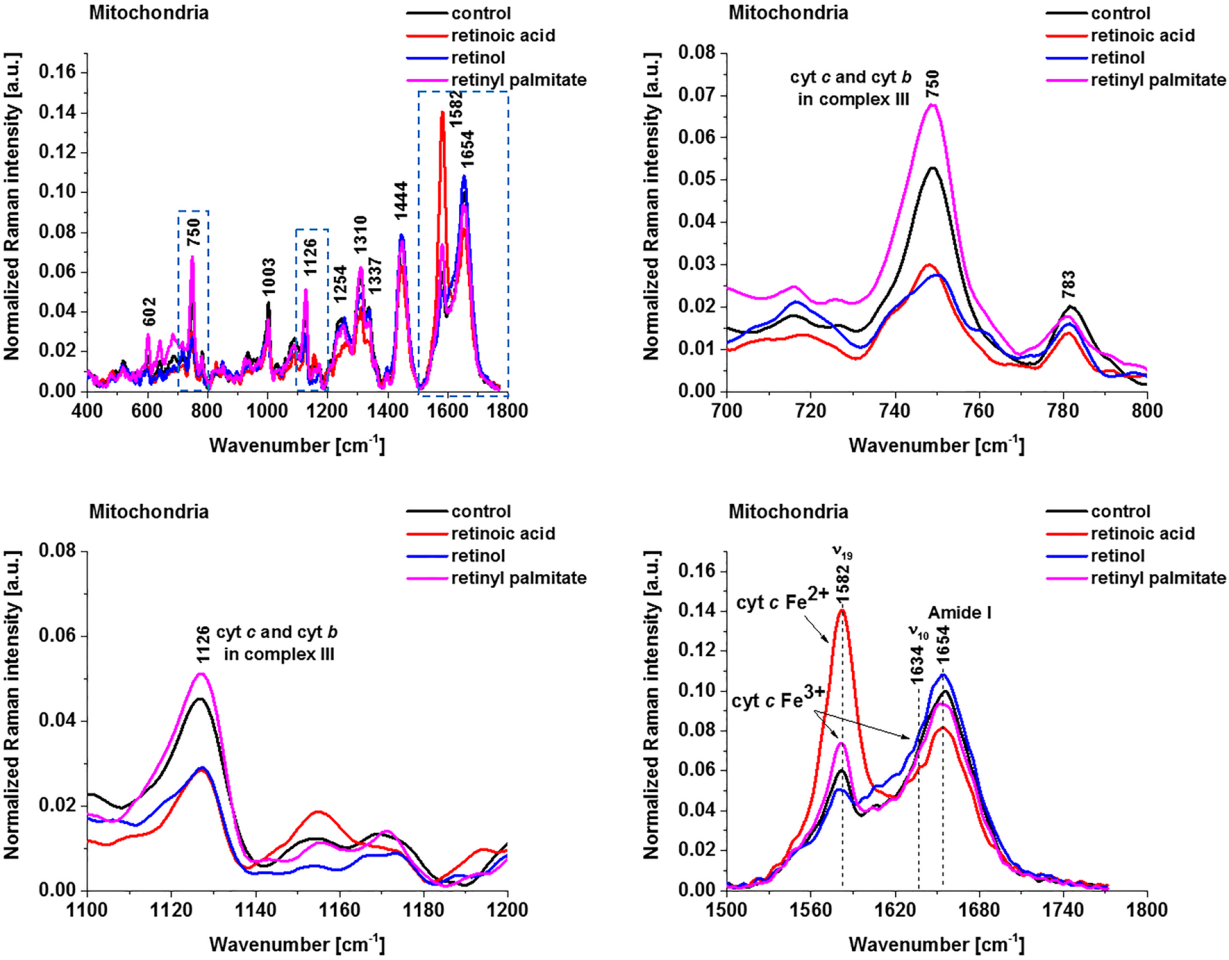
Normalized average Raman spectra of mitochondria of breast cancer cells (SK-BR-3) incubated with retinoic acid, retinol and retinyl palmitate (50 μM for 24 h). Control (black), retinoic acid (red), retinol (blue) and retinyl palmitate (magenta).

In the view of the results presented so far it is important to answer if other members of the retinoid family have also such a spectacular effect on the redox status of cytochrome *c*. Figure 4 shows Raman spectra of mitochondria for breast cancer cells SK-BR-3 (control) and SK-BR-3 incubated with retinoic acid, retinol and retinyl palmitate. One can see that for breast cancer cell incubated with retinoic acid the Raman signal of the ν_19_ vibration corresponding to cytochrome *c* in mitochondrion^13^ is significantly stronger than that for incubation with retinol and retinyl palmitate, which are similar to the Raman signal of the control cell. In contrast, for the vibrations at 750 cm^−1^ and 1126 cm^−1^ corresponding to mixed contribution from both cytochrome *c* and cytochrome *b* the Raman signals decrease for the breast cancer cell incubated with retinoic acid and retinol compared to the control cell and retinyl palmitate. It indicates that the contribution from cytochrome *b* dominates over cytochrome *c*. Second, the amount of oxidized cytochrome *b* is higher in mitochondria of cells incubated with retinoic acid and retinol compared to the control and retinyl palmitate.

To understand mechanisms responsible for change of the redox status upon incubation with retinoids and differences between vibrations of cytochrome *c* and cytochrome *b* presented in Figs. 3 and 4 let us briefly remind the role of cytochrome *c* and cytochrome *b* in the electron transfer chain that play a key role in oxidative phosphorylation. The cytochrome *b* is located in *bc1* complex III. Each cytochrome contains a heme group with an iron ion in the center, which, when receiving an electron, changes from Fe^3+^ to Fe^2+^ oxidation state. After donating the electron to the next carrier, the iron atom returns to the Fe^3+^ state. The cytochrome *bc1* complex transfers electron to cytochrome *c*, which in turn transfers it to cytochrome oxidase (complex IV) containing two cytochromes (cytochrome *a* and cytochrome *a3*) bound to two copper atoms (Cu A and Cu B), respectively. During electron transfer, the copper atoms oscillate between the Cu^2+^ state and the Cu^1+^ state.

Deeper inside into the mechanisms responsible for change of the redox status upon incubation with retinoids and differences between vibrations of cytochrome *c* and cytochrome *b* presented in Figs. 3 and 4 for mitochondria can be obtained by analysis of other organelles. Generally, more information can be obtained from the low frequency region (fingerprint) than from that of the high frequency region.

**Figure 5:**
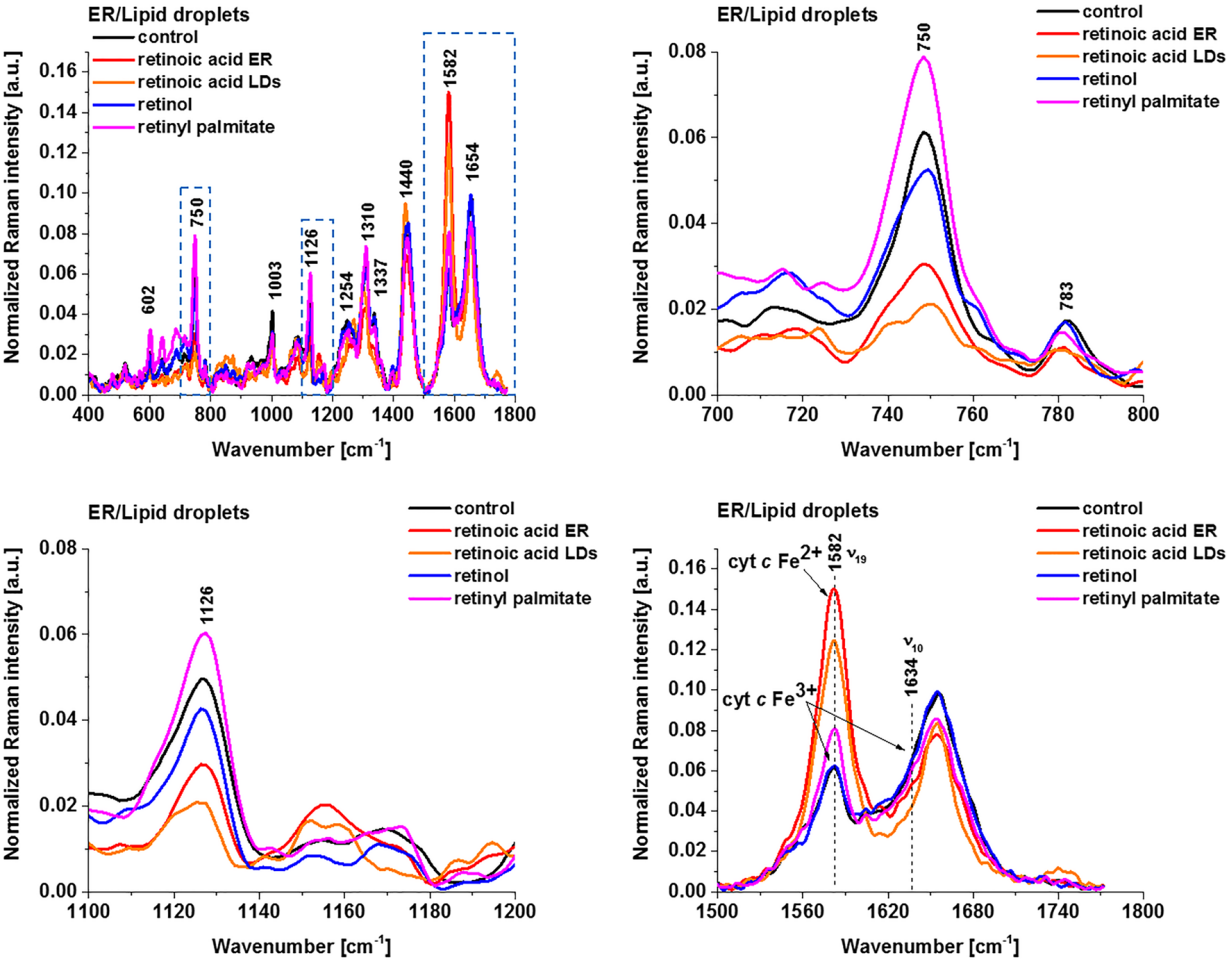
Normalized average Raman spectra of lipid droplets and endoplasmic reticulum of breast cancer cells (SK-BR-3) incubated with retinoic acid, retinol and retinyl palmitate (50 μM for 24 h). Control (black), retinoic acid (red/orange), retinol (blue) and retinyl palmitate (magenta).

Figure 5 shows the average Raman spectra of cytochromes in lipid droplets and endoplasmic reticulum of breast cancer cells SK-BR-3 (control) and SK-BR-3 incubated with retinoic acid, retinol and retinyl palmitate.

The results are similar to those in mitochondria presented in Fig. 4. Breast cancer cells incubated with retinoic acid results in significant enhancement of the Raman signals of the ν_19_ vibration corresponding to cytochrome *c* in lipid droplets and endoplasmic reticulum, which are significantly stronger than those for retinol, retinyl palmitate and control cells.

**Figure 6:**
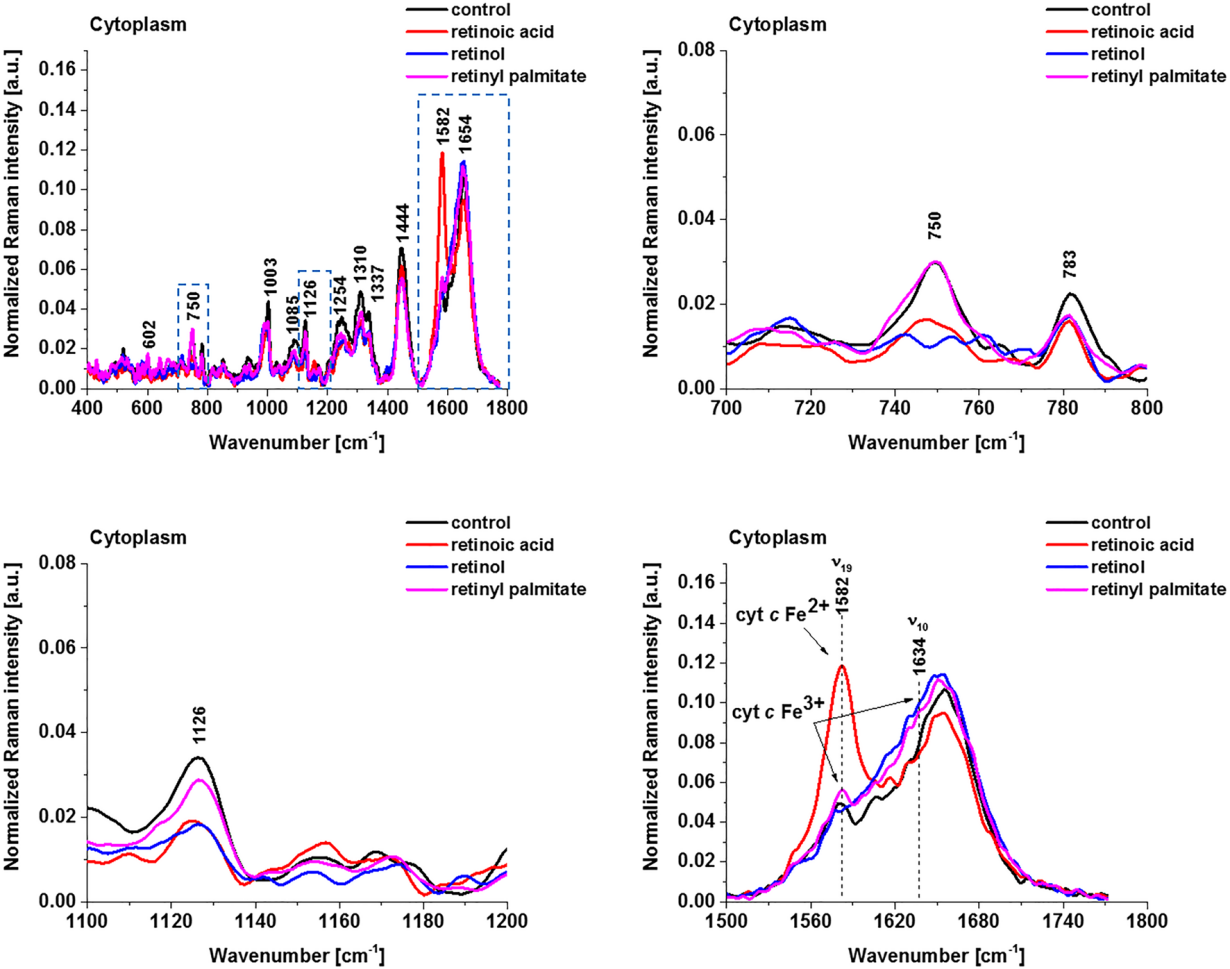
Normalized average Raman spectra of cytoplasm of breast cancer cells (SK-BR-3) incubated with retinoic acid, retinol and retinyl palmitate (50 μM for 24 h). Control (black), retinoic acid (red), retinol (blue) and retinyl palmitate (magenta).

Figure 6 shows Raman spectra of cytochromes in cytoplasm of breast cancer cells SK-BR-3 (control) and SK-BR-3 incubated with retinoic acid, retinol and retinyl palmitate.

Figure 6 shows enhanced release of the cytochrome *c* to cytoplasm induced by retinoic acid. Indeed, the Raman intensity at 1582 cm^−1^ dramatically increase upon incubation of human breast cancer cells with retinoic acid when compared with retinol, retinyl palmitate and control sample. The results from Fig. 6 shows that retinoic acid stimulates the release of cytochrome *c* from mitochondria, suggesting a potential target of retinoids in the induction of cell apoptosis, and suggest that retinoids can be considered as inducers of permeability transition. At present, the specific mechanism of action of retinoic acids on mitochondrial metabolism and in particular on the induction of permeability transition is not clear. Mitochondrial permeability transition and release of cytochrome *c* induced by retinoic acids was previously suggested by Rigobello et al.^20^.

We showed that retinoic acid and to lesser extent retinol affect the redox state of heme of cytochrome *c* and cytochrome *b* in mitochondria. The change of the redox status of cytochrome *c* and cytochrome *b* from the oxidized form to the reduced one in the electron transfer chain located in mitochondria has very serious consequences resulting in inhibition of activation apoptosis and cytokine induction. Therefore, cytochrome *c* cannot play the role of a DAMP able of alarming the immune system of danger in cells or tissue by alterations in conventional mitochondrial signaling pathways.

**Figure 7:**
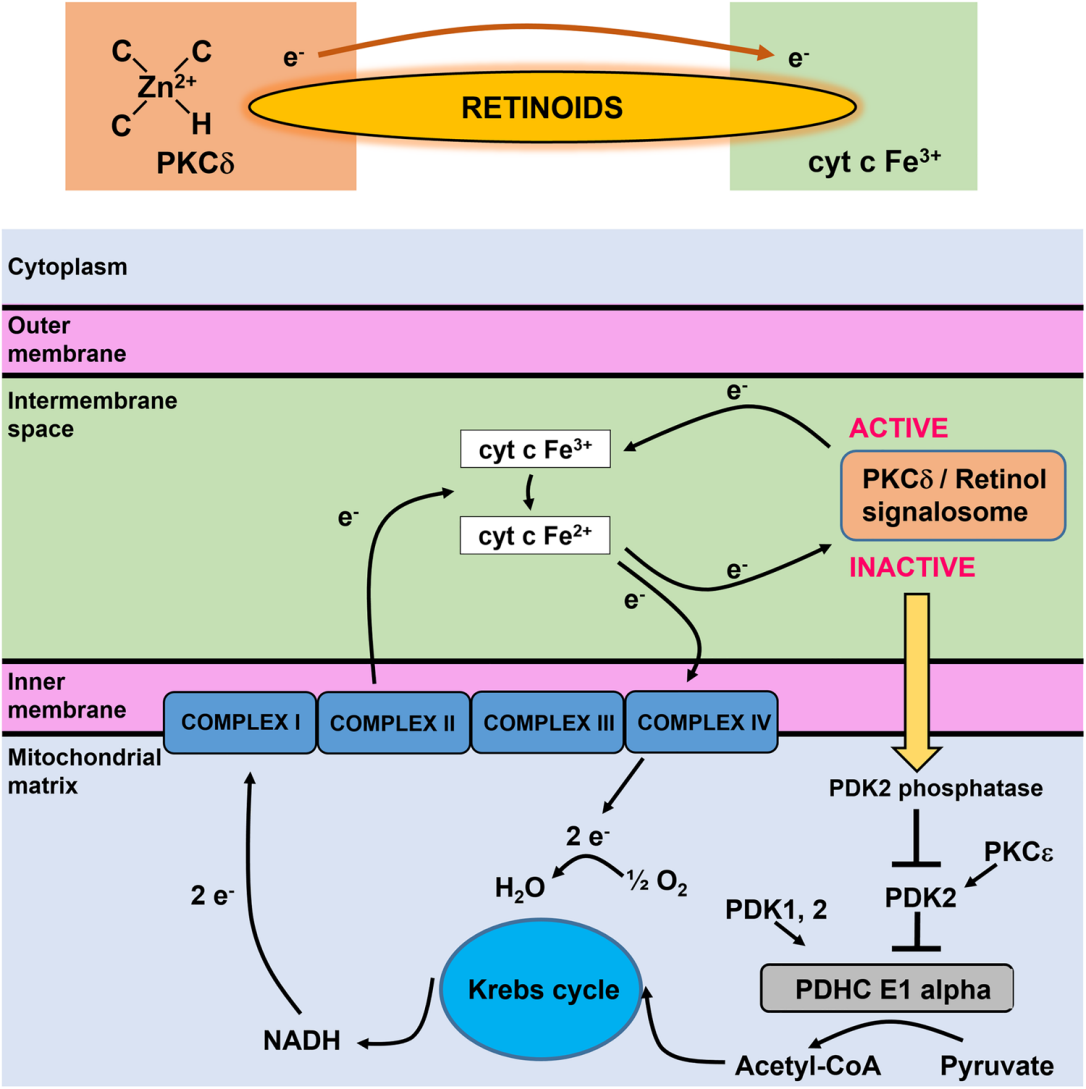
Mechanism of reversible redox regulation by the PKCδ/Retinol signalosome, located in the mitochondrial intermembrane space based on Hammerling^21^.

To explain the effect of retinoids on the redox status of cytochrome *c* in the electron transfer chain we used the quantum chemistry models of retinoid biology^21^. It has been proposed how retinol catalyzes resonance energy transfer (RET) reactions^19^. The paper suggests that RET is pivotally important for mitochondrial energy homeostasis by controlling oxidative phosphorylation. The key role in this process is played by protein kinase C δ (PKCδ). PKCδ triggers signal to the pyruvate dehydrogenase complex. The PKCδ–retinol complex reversibly responds to the redox potential of cytochrome *c*, that changes with the electron transfer chain flux (Fig. 7).

To understand the role of retinoids in cellular signal transduction we combined our results presented so far with the mechanism of reversible redox regulation by the PKCδ/Retinol signalosome presented in Fig. 7. The model proposed by Hammering et al.^21^ was derived originally for PKCδ/Retinol, but our results suggest the mechanism is even more effective for PKCδ/Retinoic acid. The PKCδ/Retinoids complex is located in the mitochondrial intermembrane space. The PKCδ signal complex consists of the protein kinase Cδ, the adapter protein Src homologous-collagen homolog (p66Shc), cytochrome *c* and retinoic acid. Retinoid molecule binds PKCδ on a site in close proximity of its zinc-coordinated structure. It was suggested in Ref.^21^ that a conserved tryptophane residue enables resonance energy transfer^22^ from PKCδ to retinoid molecule. The retinoid molecule is believed to catalyze electron (or rather energy) transfer to cytochrome *c* (Fe^3+^), initiating an unfolding process that yields the active form of the enzyme. The electron transfer from PKCδ via retinoid molecule to oxidized cytochrome *c* (Fe^3+^) makes cytochrome *c* reduced (Fe^2+^).

Inactivation of PDK2 by the PKCδ/Retinoid molecule signalosome enhances the activity of pyruvate dehydrogenase phosphatases (PDK1,2) that effectively dephosphorylates, and thereby activates, the E1 alpha regulatory domain of PDHC, leading to enhanced production of Acetyl-CoA from Pyruvate. Acetyl-CoA enters the Krebs cycle, driving the generation of NADH and increased electron transfer to complex III containing both cytochrome *c* as well as cytochrome *b*. The increased electron flux to complex III gradually replace the channel of signalosome-associated reduction of oxidized cytochrome *c*, bringing about a reversal of redox polarity, and thus enabling the passage of electrons back to oxidized PKCδ. As the reduced, inactive form of PKCδ is restored, signaling to PDHC is weakened and consequently fuel flux diminishes, reducing the risk of overload in electron transfer chain.

We suggest that the process of reversible redox regulation the PKCδ/Retinoic acid (and to a lesser extent PKCδ/Retinol) signalosome occurs only at normal physiological conditions. In contrast, during the cancer development the process of resonance energy transfer (RET) irreversibly activates PKCδ. Consequently, while capable of triggering the exergonic activating pathway, retinoids fails to activate the endergonic silencing path, trapping PKCδ in the ON position and causing harmful levels of reactive oxygen species.

This theoretical hypothesis presented in Ref.^19^ received experimental support from our results presented in the paper. All Figures show enhanced production of reduced Fe^2+^ cytochrome *c*. The enhanced electron flux to cytochrome *c* from unbalanced PKCδ/Retinoid molecule signalosome increases spectacularly the Raman signal of the reduced cytochrome in cancer cells, because the reduced cytochrome (Fe^2+^) has much higher intensity of the Raman bands than the oxidized cytochrome *c* (Fe^3+^)^13^. Consequently, the rise in Raman signal at 1582 cm^−1^ following incubation with retinoic acid suggests a conversion from oxidized cytochrome *c* to reduced cytochrome *c*.

The switch from reversible to irreversible RET mechanism in cancers is unclear, but we suggest that it may be related to the apocarotenoids, the primary products of the mitochondrial β-carotene, 9′-10′-oxygenase. It was reported by Hammerling et al.^21^ that apocarotenoid have specific features, including irreversible modulation of energy homeostasis.

**Figure 8:**
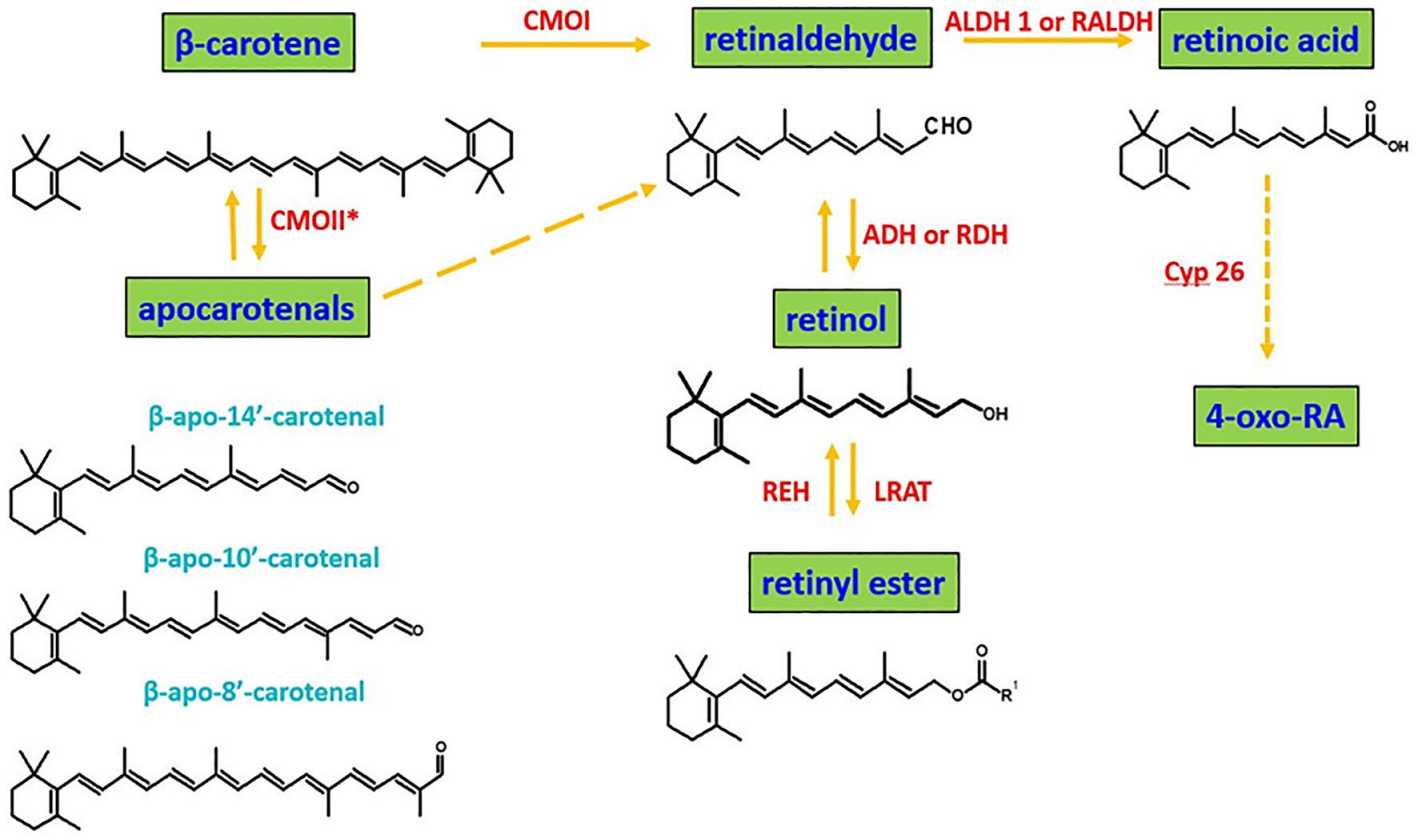
Summary of β-carotene, retinoids, and cytochromes metabolism^23, 24^.

To discuss the role of retinoic acid on cancer development we must take into account the fact that the concentration of retinoic acid in human tissue depends on a few families of enzymes presented in Fig. 8. First, β-carotene is transformed by CMOII enzyme into apocarotenals. Second channel is believed to be more important for cancers. β-carotene is cleaved by an enzyme of β-carotene 15,15′-oxygenase (CMOI) into two molecules of retinal (retinaldehyde)^23^. Retinal is catalysed by the alcohol dehydrogenase (ADH) family to generate retinol, LRAT converts retinol into retinyl esters. Retinal catalysed by RALDH enzyme forms retinoic acid (RA) (transcriptionally active), which can metabolize by enzymes that belong to the cytochrome P450 (CYP) 26 family into more polar compounds, including 4-oxo retinoic acid, which are believed to be transcriptionally inactive^24^.

It is possible that competition between CMOI and CMOII enzymes decide about reversibility or irreversibility of RET mechanism in cancers.

## Conclusions

In the context of the current state of knowledge on impact of retinoids on human breast adenocarcinoma, we demonstrated by Raman imaging that retinoids regulate the redox status of cytochrome *c* in cancer cells. We showed that Raman imaging is effective assay for detecting redox status of cytochrome *c* in specific cell organelles upon incubation with retinoic acid, retinol and retinyl palmitate. Therefore, in contrast to existing analytical technologies Raman imaging can detect the full extent of cytochrome *c* localization inside and outside specific organelles. In Raman imaging we do not need to disrupt cells to open them to release the cellular structures to learn about their biochemical composition.

We studied impact of retinoids on the redox status of the central iron ion in heme of cytochrome *c*. We determined the redox status of cytochrome *c* in mitochondria, cytoplasm, lipid droplets, and endoplasmic reticulum of the human breast cancer cells. We recorded the Raman spectra and images in human breast cancer in vitro cells receiving redox stimuli by retinoic acid, retinol and retinyl ester (palmitate). We incubated human breast cancer cells (adenocarcinoma SK-BR-3) with retinoic acid, retinol and retinyl ester (palmitate) at concentration of 50 μM for 24 h of incubation. We discussed the role of retinoic acid in oxidative phosphorylation and signaling of cancer cells. We showed that retinoic acid and to a lesser extend retinol is pivotally important for mitochondrial energy homeostasis by controlling the redox status of cytochrome *c* in the electron transport chain controlling oxidative phosphorylation and apoptosis.

We found that retinoic acid spectacularly modifies mitochondrial functional activity of cancer cells that is monitored by the Raman spectroscopy and imaging. Abnormal retinoic acid and retinol signaling in the mitochondria, cytoplasm, lipid droplets, endoplasmic reticulum and nucleus monitored by Raman signal at 1582 cm^−1^ has been demonstrated in human cancer cells. These findings are promising to support the direct study of changes of the redox status of cytochrome *c* in mitochondria (from the oxidized to reduced form), considering that the reduced form has very serious consequences resulting in inhibition of respiration, apoptosis and cytokine induction.

The paper provides experimental support for theoretical hypothesis how retinoic acid/retinol catalyse resonance energy transfer reactions and controls the activation/inactivation cycle of protein kinase PKCδ.

## Materials and methods

### Reference chemicals

Cytochrome *c* (no. C2506) and retinoic acid (no. R2625), retinol (no. R7632) and retinyl palmitate (no. R3375) were purchased from Merck Life Science.

### Sample preparation

#### Cell culture and preparation for Raman spectroscopy and fluorescence imaging

The studies were performed on a human breast adenocarcinoma SK-BR-3 cell line (no. HTB30, ATCC) which amplifies and overexpresses the HER-2/neu oncogene and expresses the HER-3, HER-4 and p53 oncogenes. SK-BR-3 cells were grown in ATCC-formulated McCoy’s 5a medium (no. 30-2007, ATCC) with 10% fetal bovine serum (FBS no. 30-2020, ATCC) and maintained at 37 °C in a humidified atmosphere containing 5% CO_2_. Cells were seeded on a CaF_2_ window (Crystran Ltd., Poole, UK; CaF_2_ Raman grade optically polished window 25 mm diameter × 1 mm thick, no. CAFP25-1R, Poole, UK) in a 35 mm Petri dish at a density of 5 × 10^4^ cells per Petri dish the day before the examination. Before Raman examination, cells were supplemented with retinoic acid, retinol and retinyl ester (palmitate) at concentration of 50 μM by 24 h, then fixed with 4% formalin solution (neutrally buffered) and kept in PBS (no. 10010023, Gibco) during the experiment. After conducting Raman imaging measurements, the cells were treated with Hoechst 33342 (25 μL at 1 μg/mL per mL of PBS) and Oil Red O (10 μL of 0.5 mM Oil Red dissolved in 60% isopropanol/dH_2_O per each mL of PBS) by means of a 15 min incubation. Following a PBS wash, the cells were imaged for fluorescence using an Alpha 300RSA WITec microscope, with the addition of fresh PBS.

#### Raman spectroscopy and imaging

Raman measurements of the human breast adenocarcinoma were conducted on a WITec confocal alpha 300 Raman microscope with the use of 532 nm excitation wavelength coupled to the microscope via an optical fiber (50 μm diameter). The 40 × objective (NIKON CFI Plan Fluor C ELWD (Extra-Long Working Distance) 40 ×: N.A. 0.60, W.D. 3.6–2.8 mm; DIC-M, C.C.0-2) was used. A standard alignment procedure (single-point calibration) was performed before the collection of Raman spectra with the use of Raman scattering vibration produced by a silicon plate (520.7 cm^−1^). The spectra were measured with 532 nm excitation wavelength laser with the power of 10 mW in the focus spot and with an integration time of 0.3 s by Andor Newton DU970-UVB-353 CCD camera in enhanced mode (EMCCD). Raman images were recorded with a spatial resolution of 1 × 1 µm. A typical Raman map of a single cell consists of 1400 Raman spectra (map size 40 × 35 µm). Raman data analysis was performed using WITec (WITec Project Plus 4) and OriginPro 2018 programs. Raman imaging data were analyzed by the Cluster Analysis method described in our previous papers^14,15^. Number of analyzed cells n(SK-BR-3) = 11, n(SK-BR-3 with retinoic acid) = 5, n(SK-BR-3 with retinol) = 4, n(SK-BR-3 with retinyl palmitate) = 7; number of control and incubated with retinoic acid, retinol, retinyl palmitate Raman spectra used for averaging 18,125, 8525, 8325 and 6825, respectively.

### Supplementary Information

Supplementary Figures.

## Data Availability

The datasets generated and analyzed during the current study are available from the corresponding author on reasonable request.
