# Supplementary material for: Confocal Raman imaging reveals the impact of retinoids on human breast cancer via monitoring the redox status of cytochrome c
Source: Sci Rep. 2023 Sep 12;13:15049. doi: 10.1038/s41598-023-42301-z (PMC10497563; doi:10.1038/s41598-023-42301-z)
Supplement: Supplementary file 1 — Supplementary Figures. [file 41598_2023_42301_MOESM1_ESM.docx]

**Supplementary information**

**
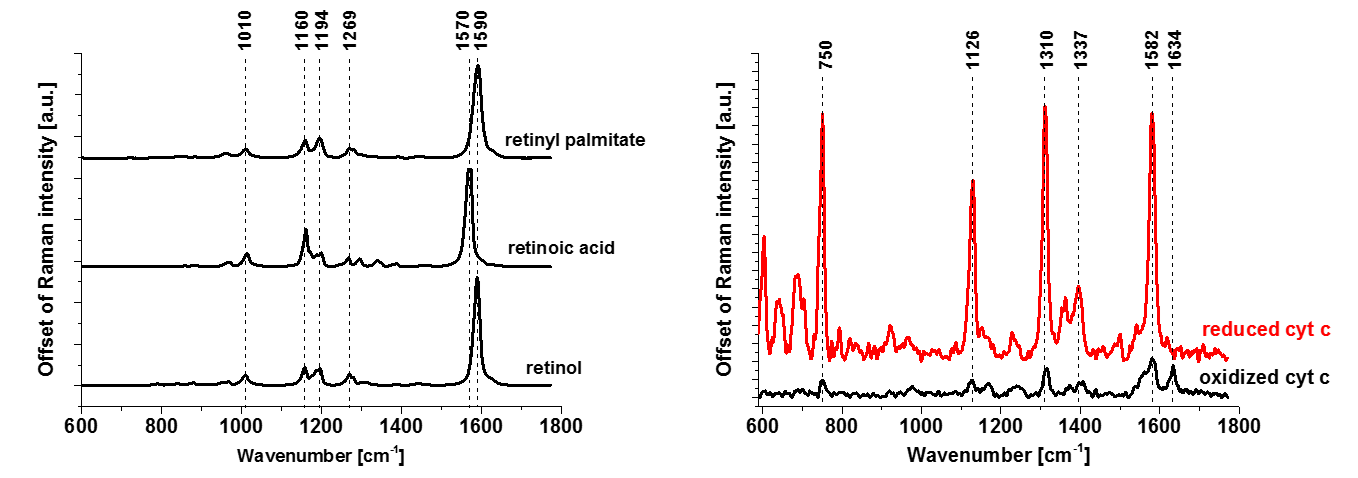
**

**Supplementary Figure 1.** Raman spectra of retinol, retinoic acid, retinyl palmitate, oxidized and reduced form of cytochrome c (0.46 mM of cytochrome *c* in PBS solutions, reduction agent NaBH_4_ in tenfold excess). 532 nm, 10 mW, 0.5 second, 10 accumulations.


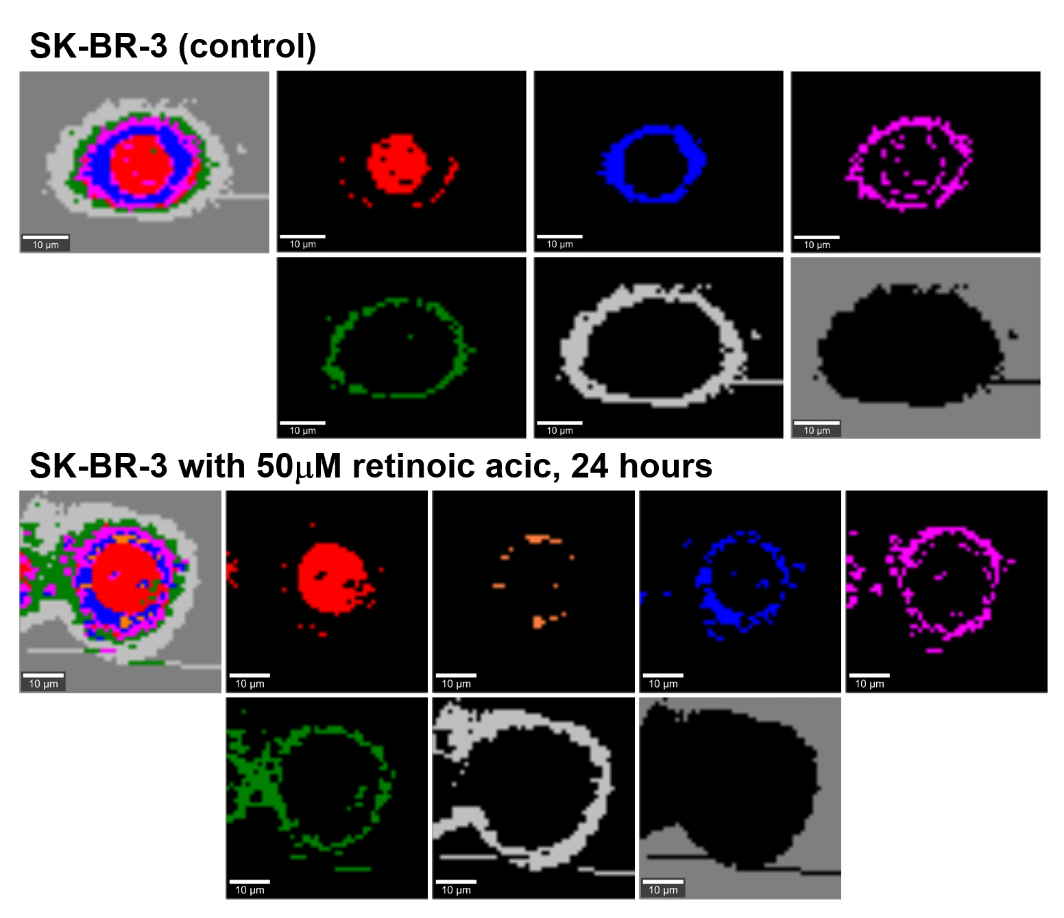


**Supplementary Figure 2.** Raman images and individual clusters of SK-BR-3 (without and with supplementation of 50 μM retinoic acid). White scale bar of 10 μm.
